# Threshold estimation in running using dynamical correlations of RR intervals

**DOI:** 10.14814/phy2.70241

**Published:** 2025-05-09

**Authors:** Matias Kanniainen, Vesa Laatikainen‐Raussi, Teemu Pukkila, Krista Vohlakari, Esa Hynynen, Johanna K. Ihalainen, Esa Räsänen

**Affiliations:** ^1^ Computational Physics Laboratory Tampere University Tampere Finland; ^2^ Faculty of Sport and Health Sciences University of Jyväskylä Jyväskylä Finland; ^3^ Finnish Institute of High Performance Sport KIHU Jyväskylä Finland

**Keywords:** aerobic threshold, anaerobic threshold, exercise physiology, heart rate variability

## Abstract

We study the estimation of aerobic threshold (AeT) and anaerobic threshold (AnT) using dynamical detrended fluctuation analysis (DDFA). Conventionally, the thresholds are estimated in laboratory settings, where the subject performs an incremental exercise test on a cycloergometer or treadmill. We compared DDFA‐based thresholds (DDFAT_1_ and DDFAT_2_) with lactate thresholds (LT_1_ and LT_2_) and examined thresholds derived from theoretical and measured maximal heart rates (HR). The analysis was conducted on 58 subjects undergoing an incremental treadmill running test. Our findings indicate significant discrepancies between thresholds derived from theoretical and measured maximal HRs compared to lactate thresholds. Specifically, theoretical maximal HR thresholds consistently underestimated lactate thresholds, exhibiting systematic bias. Measured maximal HR thresholds also showed a consistent underestimation, though with improved alignment to lactate thresholds. In contrast, the DDFA‐based method demonstrated reasonable agreement with lactate thresholds and lacked systematic bias. The DDFA‐based approach offers a simple and accurate alternative for estimating AeT and AnT. Its potential for continuous monitoring makes it suitable for integration into wearable devices such as smartwatches and heart rate monitors.

## Graphical abstract

We utilize dynamical detrended fluctuation analysis (DDFA) for RR interval time series to evaluate the exercise intensity and estimate the aerobic and anaerobic thresholds. Changes in the dynamical correlations of the heart rate variability during exercise provide a promising non‐invasive way to estimate the aerobic and anaerobic thresholds in running.

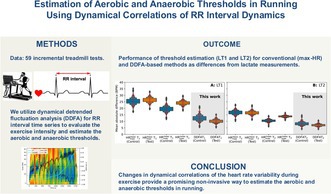

## 1 INTRODUCTION

Training intensity in endurance sports can be described using a three‐zone exercise model (Stöggl & Sperlich, 2019), where the exercise physiological thresholds separate the zones. The two thresholds are referred to as the aerobic threshold (AeT) and anaerobic threshold (AnT), respectively. For endurance athletes, understanding these thresholds is crucial for optimizing training. Training continuously at too high an intensity increases the risk of injuries and overtraining due to excessive physical stress.

Conventionally, the thresholds are determined from the lactate concentration (LT_1_, LT_2_), or from the ventilatory gas exchange (VT_1_, VT_2_) (Binder et al., 2008). LT_1_ is defined as the point where the rate of lactate production becomes greater than the rate of lactate clearance in the body, but is still able to reach a steady state for the current exercise work rate. LT_2_, on the other hand, corresponds to the point where the lactate production overcomes the lactate clearance, and steady state in the production/clearance is not achievable any more even though the exercise rate is steady (Faude et al., 2009). The lactate thresholds correspond to changes in metabolic activity caused by the exercise intensity, but the determination of the lactate thresholds is often costly, cumbersome and time‐consuming, so the measurements are not carried out regularly except for professional endurance athletes. The lactate thresholds are often determined by a professional exercise physiologist in laboratory settings. However, the determination of the thresholds is subjective and ambiguous due to the different metabolic responses and initial lactate levels of the individuals, and there is no universal method to be referred as the golden standard (Jamnick et al., 2018; Newell et al., 2007).

On the other hand, ventilatory thresholds correspond to the changes in the cardiopulmonary activity. Akin to lactate thresholds, the determination of VTs also requires a specialized test environment with strict quality control and trained personnel (Cannon et al., 2009; Gaskill et al., 2001). Although VTs are closely related to and correlate with LTs, they do not correspond quantitatively (Cerezuela‐Espejo et al., 2018). This has led to controversy over the methodologies used to study the well‐defined physiological and metabolic changes that occur during exercise. Consequently, there is a growing need for standardized methods that more accurately reflect the physiological changes at these thresholds (Chavez‐Guevara et al., 2024; Sperlich & Gronwald, 2024).

Heart rate (HR) and HR variability (HRV) measurements are becoming increasingly more popular due to enhanced signal quality of the wearable devices such as smartwatches. This allows a variety of possibilities for analysing physiological signals, such as electrocardiogram (ECG) and RR interval (RRI) series during exercise. Current smartwatches have several measures to determine physiological changes, for example, the training zones during exercise based on HR or HRV measurements with varying reliability and applicability (Campen et al., 2020; Cottin et al., 2006). Several wearable devices utilize the estimated maximal HR (HR_max_) to assess the training zones. Typically, the first HR threshold (HR_max_T_1_) is estimated to be around 60%–70% of the HR_max_, and the second HR threshold (HR_max_T_2_) around 80%–90% of the HR_max_, respectively (Marx et al., 2018). It is still widely used in fitness and exercise applications, even though it has gained considerable criticism (Colantonio & Peduti Dal Molin Kiss, 2013; Robergs & Landwehr et al., 2002). The estimates have been shown to fail to account for the population‐wide individual variability and therefore provide unreliable results for, for example, highly trained athletes (Faff et al., 2007; Shookster et al., 2020). Furthermore, the relative percentages of the maximal HR associated with the physiological thresholds are strongly individual.

HRV describes the variability between successive heart beats, that is, interbeat intervals (Goldberger, 2020). HRV methods can be used to analyse the physiological response to both rest and exercise (Gronwald & Hoos, 2020). There are several different HRV metrics based on different approaches (Shaffer & Ginsberg, 2017). For example, the frequency‐domain methods have been used for threshold estimation in multiple studies (Di Michele et al., 2012; Ramos‐Campo et al., 2017). However, in recent years, nonlinear HRV metrics have received considerable attention in sport applications. For example, the short‐term scaling exponent *α*_1_ of detrended fluctuation analysis (DFA) (Peng et al., 1995)—which describes the characteristic correlations of the RRI series (see below)—has shown potential in the assessment of exercise load and intensity, as well as the thresholds compared to the ventilation measurements (Gronwald et al., 2021; Rogers et al., 2021).

In this study, we utilize an extension of DFA, that is, *dynamical* detrended fluctuation analysis (Molkkari et al., 2020) (DDFA), to estimate the AeT and AnT. The DDFA algorithm expands the analysis of DFA by calculating the scaling exponents dynamically as functions of both time *t* and scale *s*, thus providing a comprehensive view of the changing physiological conditions during exercise. Increased exercise intensity has been shown to decrease the scaling exponent values for both DFA and DDFA (Rogers et al., 2021; Gronwald et al., 2021; Kanniainen et al., 2023). The algorithm for estimating the physiological thresholds with DDFA was first introduced in (Kanniainen et al., 2023), where the thresholds were calculated for 15 subjects during a cyclo‐ergometer test and compared to conventional methods. The present study serves as the further validation for the method with a larger dataset of incremental running treadmill exercise tests. There are significant physiological differences in cycling and running; for example, the muscle pump efficiency, the ventilatory response, and the heart rate levels for maximal and submaximal intensities are different between the two (Millet et al., 2009). Furthermore, the biomechanics of running and cycling are fundamentally different due to the use of different muscle groups, and the exercise settings such as cadence and treadmill incline have an effect on the respiratory rate and heart rate variability of the subjects during exercise (Lunt et al., 2011). Therefore, it is important to study the validity of the method based on the RR interval correlations irregardless of the exercise mode. We calculate the first and second DDFA‐based thresholds (DDFAT_1_ and DDFAT_2_), respectively, and compare them against the lactate thresholds determined by an experienced exercise physiologist. We also evaluate the validity of the thresholds determined from the maximal HR, which are conventionally used in the wearable devices to estimate the training zones. We show that the previously consistent results in cycloergometer tests are also valid in treadmill running tests, and the results are relatively comparable to the lactate thresholds. Furthermore, we study the relationship between the behavior of the DDFA scaling exponents and lactate concentration in detail.

## 2 METHODS AND DATA

### 2.1 Participants

For this study, 71 participants were recruited as part of a larger study conducted in the University of Jyväskylä. Data collection was performed during an ongoing study project, “Performance variation and health monitoring of recreational runners,” that monitors training, health, and recovery of recreational and endurance runners for one calendar year. The study protocol was approved by the ethics committee of the University of Jyväskylä (534/13.00.04.00/2023). Participants signed a written informed consent to participate in the study project. The participants had a very different training background, where some of the participants did occasional recreational exercise and some participants had a background in regular endurance exercise. All of the participants performed a maximal V̇O2 test (Buttar et al., 2019). The information for sex, age, maximal heart rate, maximal V̇O2 intake, maximal lactate concentration, maximal reached speed, and the test duration is shown in Table 1, which includes only the subjects who were remaining after the preprocessing (see Section 2.3).

**TABLE 1:** Statistical information of the participants, as mean value ± standard deviation: Sex (male = M, female = F), age (years), maximal heart rate HR_max_ (BPM), maximal V̇O2 intake V̇O2_max_ (mL/kg/min), maximal lactate concentration Lactate_max_ (mmol/l), maximal reached speed Speed_max_ (km/h), and the test duration (min:sec).

Variable	Studied subjects
Sex (M/F)	31/27
Age	33 ± 8
HR_max_ (BPM)	192 ± 9
V̇O2_max_ (mL/kg/min)	47.1 ± 8.0
Lactate_max_ (mmol/l)	10.0 ± 2.2
Speed_max_ (km/h)	14.5 ± 2.3
Test duration (s)	24:09 ± 4:06

### 2.2 Test protocol

The performed exercise test was an incremental treadmill test with the H/P/Cosmos Saturn 300/100 r treadmill with a constant incline of 1%, and the speed of the treadmill was increased every 3 min by 1 km/h. Ventilation parameters were measured breath‐to‐breath with Jaeger VyntusTM CPX. Lactate concentration was measured from capillary blood samples from the fingertip and analysed with the Biosen S‐line Lab+ lactate analyser before the first stage and after each stage. The treadmill was shortly paused after each level to obtain the blood sample.

Starting speed was determined individually according to the assessment of the study physiologist and possible previous threshold test history. Starting speed was estimated to be 2–3 km/h below the aerobic threshold aiming at total test duration of less than 30 min when test is completed until exhaustion.

### 2.3 Preprocessing

The exercise measurements included a short warm‐up period, the exercise, and the recovery phase. The measurements were divided manually so that only the exercise phase was considered, beginning with the starting level and ending with the highest level reached at the end of the training. The RR intervals were preprocessed to ensure good data quality for the analysis. First, the RRI time series was filtered with a rolling median filter, with a window of 21 beats. The RRIs which deviated from the median by more than ±5% were discarded. In addition, the RRI values deviating more than 200 ms from the preceding RRIs were discarded. The recordings of the subjects for which 5% or more of the intervals were filtered were discarded from the analysis. Furthermore, there was one sample for which our method could not determine the DDFAT_2_, even though the LT_2_ and HR_max_T_2_s were found. This sample was omitted not to neglect the error of our method in determining the threshold for the sample in question, but to be able to directly compare the resulting DDFATs, LTs, and HR_max_Ts for all tested subjects. This left us with the exercise tests of 58 subjects.

### 2.4 Lactate thresholds

The lactate thresholds were defined by the study physiologist (VLR), and any ambiguous cases were examined carefully by VLR and EH. The aerobic threshold was determined based on a 0.3 mmol/L increase from the lowest measured reading of the test, and the anaerobic threshold using the intersection of two fitting lines. The first fitting line was determined according to the lactate value of the load before and after the aerobic threshold, and the second fitting line based on each lactate value that increased by more than 0.8 mmol/L from the previous load. Analyses were performed using K‐lab 3.1.25 software and adjusted subjectively by study physiologist (VLR) if needed.

### 2.5 Heart rate and heart rate variability thresholds

#### 2.5.1 Maximal HR‐based thresholds

We calculated the thresholds based on maximal HR analysis. In the analysis, we calculated the theoretical age‐dependent maximal HR with the conventional formula of ${\mathrm{HR}}_{\max}^{\text{theor}}$= 220 – AGE according to (Robergs et al., 2002), but also considered the measured maximal HR values ${\mathrm{HR}}_{\max}^{\text{meas}}$ which the subjects actually reached in the exercise test. Based on both theoretical ${\mathrm{HR}}_{\max}^{\text{theor}}$ and the measured ${\mathrm{HR}}_{\max}^{\text{meas}}$ we calculated the thresholds by setting the first threshold HR_max_T_1_ at 70% of the HR_max_ value, and the second threshold HR_max_T_1_ at 85% of the HR_max_ value as in (Kanniainen et al., 2023). In Section 3 we assess the differences between the thresholds derived from the two different definitions, ${\mathrm{HR}}_{\max}^{\text{theor}}$ and ${\mathrm{HR}}_{\max}^{\text{meas}}$.

#### 2.5.2 DDFA‐based thresholds

Our method is based on DDFA (Molkkari et al., 2020) and the computational method to estimate the physiological thresholds was first introduced in (Kanniainen et al., 2023). The DDFA scaling exponents utilized in the threshold estimation are calculated as follows (Kanniainen et al., 2023; Molkkari & Räsänen, 2023):

1. Perform dynamic segmentation for each scale *s*, where the segment length *l* = 5 *s*.
2. Compute the second‐order DFA fluctuation function (Peng et al., 1995) for each segment at scales *s* − 1, *s*, *s* + 1. The logarithmic fluctuation function is thus denoted as $\tilde{F}$_t_(*s* − 1), $\tilde{F}$_t_(*s*) and $\tilde{F}$_t_(*s* + 1) at the corresponding scales.
3. In each segment, compute the dynamic scaling exponent *α*(*t*,*s*) by the finite difference approximation

$$\alpha \left(t,s\right)\approx \frac{\left[h_{-}^{2}\tilde{F_{t}}\left(s+1\right)+\left(h_{+}^{2}-h_{-}^{2}\right)\tilde{F_{t}}\left(s\right)-h_{+}^{2}\tilde{F_{t}}\left(s-1\right)\right]}{\left[h_{-}h_{+}\left(h_{+}+h_{-}\right)\right]},$$

where $h_{-}$ = log(*s*) − log(*s* − 1) and $h_{+}$ = log(*s* + 1) − log(*s*) are the logarithmic backward and forward differences.

During the computation of the time‐ and scale‐dependent scaling exponents *α*(*t*,*s*), the mean HR values of the segments can be calculated, and the scaling properties can be represented as a function of aggregated mean HR and scale, *α*(HR,*s*). This HR‐dependent scaling exponent distribution is then utilized in the determination of the thresholds as follows:

1. Calculate the second‐order DDFA (DDFA‐2) for the RRI time series with 20 logarithmically spaced integer scales between 5 and 64 RRIs. The range of scales corresponds to the joint scales of DFA *α*_1_ and *α*_2_.
2. Calculate the scaling exponents *α*(HR,*s*) as a function of HR and scale *s*, where HR corresponds to the average of each segment. Sort the HR values by binning them to the nearest integer and assign *α*(HR,*s*) values to the corresponding bins. For each scale *s*, take the mean value of the scaling exponents within the bins, which results in a distribution of *α*(HR_bin_,*s*).
3. Calculate an individual baseline to take the individual physiological starting conditions into account. First, the scaling exponents are determined for the first two speed levels corresponding to roughly 6 min of the beginning of the measurement. The lactate measurement break at the end of the second level is not considered in the baseline. A mean value of the scaling exponents for each scale is calculated and added together, thus obtaining the individual baseline value. The baseline value is then subtracted from *α*(HR_bin_,*s*) over the whole measurement.
4. Calculate the mean value of *α*(HR_bin_,*s*) over each scale *s* of a HR bin. Smoothen the resulting *α*(HR_bin_) curve with a mean filter with a kernel size of 5 HR bins to focus on the trend rather than the small local fluctuations. Thus, the mean smoothed scaling exponent $\tilde{\alpha }\left({\mathrm{HR}}_{\mathrm{bin}}\right)$ is obtained. The first DDFA‐derived threshold is the point, where $\tilde{\alpha }\left(HR_{\mathrm{bin}}\right)$ distribution drops below the baseline ($\tilde{\alpha }\left({\mathrm{HR}}_{\mathrm{bin}}\right)$ = 0). The determination of this point is derived through the following calculation. First find the points where $\tilde{\alpha }\left({\mathrm{HR}}_{\mathrm{bin}}\right)$ distribution crosses the baseline. If the intersection is not stable, that is, $\tilde{\alpha }\left({\mathrm{HR}}_{\mathrm{bin}}\right)$ keeps fluctuating around the baseline, move to the next one until a stable intersection is found. We find that sufficient stability can be found when $\tilde{\alpha }\left({\mathrm{HR}}_{\mathrm{bin}}\right)$ remains negative for at least 25 consecutive binned HR values.
5. Similarly, select the second DDFA derived threshold in a stable intersection where $\tilde{\alpha }\left({\mathrm{HR}}_{\mathrm{bin}}\right)$ equals −0.5. A stable intersection for DDFAT_2_ is found, when $\tilde{\alpha }\left({\mathrm{HR}}_{\mathrm{bin}}\right)$ remains below −0.5 for at least five consecutive binned HR values.

As in (Kanniainen et al., 2023), we utilize $\tilde{\alpha }\left({\mathrm{HR}}_{\mathrm{bin}}\right)$ to measure the physiological changes during the increasing exercise intensity. These changes provide a basis for the determination of the thresholds as described above.

### 2.6 Statistical analysis

To study the statistical properties of the estimated thresholds of different methods with respect to each other and the lactate thresholds, we calculated the Pearson's correlation coefficients *r* and their *p*‐values. (Cohen et al., 2009) We considered the one‐sided alternative hypothesis of no correlation and calculated the limits of agreement. Also, we performed the Welch's *t*‐test assuming inequal variances with the null hypothesis that the two compared groups have identical average values (Welch, 1947). Here, the *t*‐test is for independent samples, and we compare the means of the two independent groups. We also calculate sample size power of a *t*‐test for two independent groups with assuming the significance level of 0.05 and medium effect size of 0.5 of Cohen's *d* (Kelley & Preacher, 2012).

To study the agreement of the threshold estimates and the lactate thresholds, we also performed the Bland–Altman analysis (Bland & Altman, 1999). Furthermore, we considered the limits of agreement (LoA) with bias‐corrected and accelerated (BCa) bootstrapping (Efron, 1987), where the random resampling was performed 10^4^ times.

## 3 RESULTS

### 3.1 DDFA‐based threshold estimation

**FIGURE 1:**
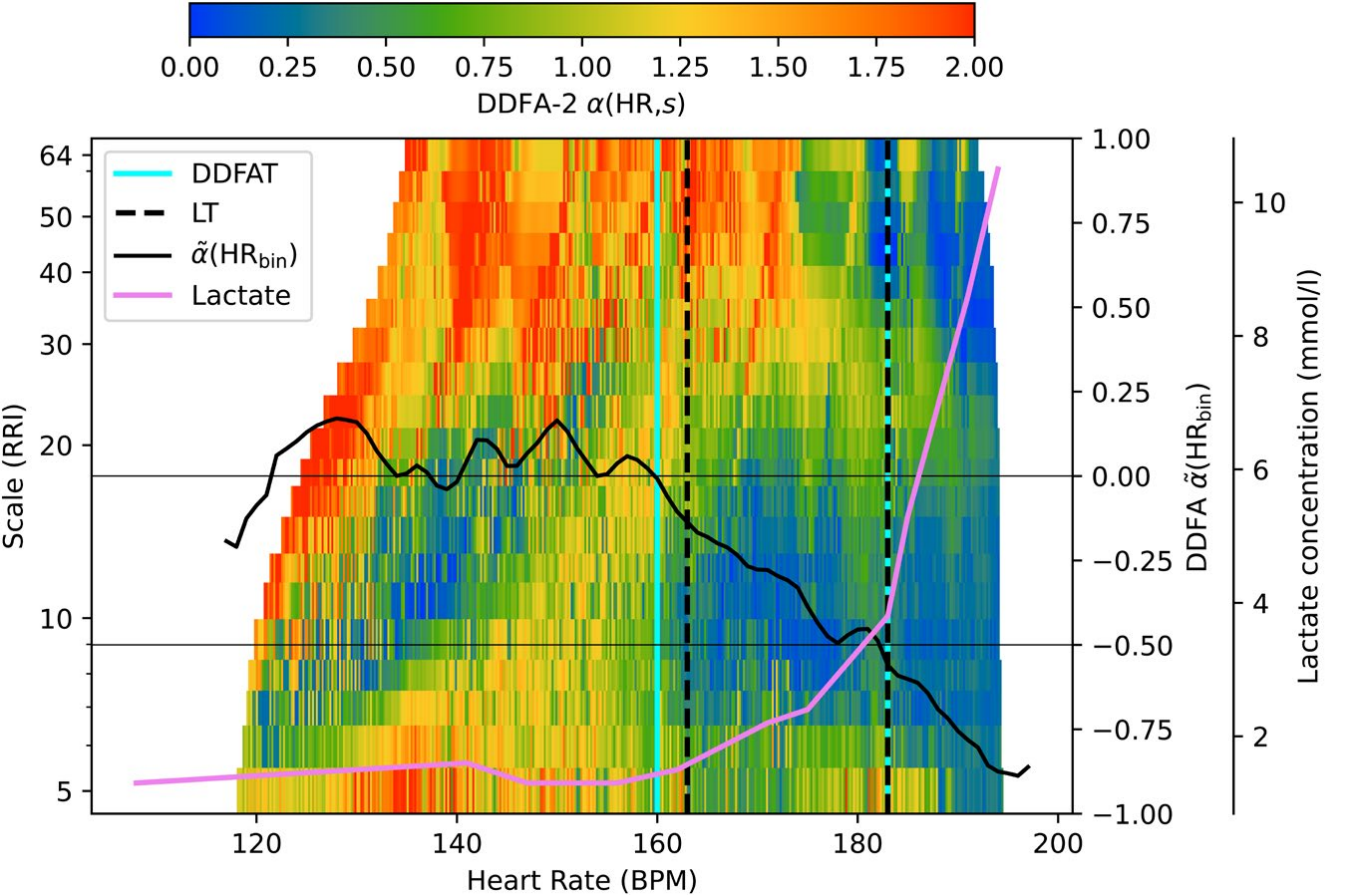
Illustration of the DDFA thresholds (cyan vertical lines) compared to the lactate thresholds (black vertical lines) as a function of binned heart rate (HR). The integrated scaling exponent $\tilde{\alpha }\left({\mathrm{HR}}_{\mathrm{bin}}\right)$ and the lactate concentration as a function of binned HR are represented with the black line and pink line, respectively.

In our method, the threshold estimation is based on dynamical changes of the DDFA scaling exponent *α*(HR,*s*). Figure 1 illustrates our method for one subject during the incremental exercise. The lactate concentration as a function of HR is shown in purple, and the colors of the plot correspond to the DDFA‐2 scaling exponent *α*(HR,*s*). As the HR is increased during the exercise, the colors turn from red (higher *α*(HR,*s*) values) to green and blue (lower *α*(HR,*s*) values). This fact is used in the determination of the thresholds. The solid black line corresponds to the $\tilde{\alpha }\left({\mathrm{HR}}_{\mathrm{bin}}\right)$ value, which is used to determine the corresponding HR values of the DDFAT_1_ and DDFAT_2_, as described in Section 2.5.2.

### 3.2 Relationship between DDFA scaling exponent and lactate concentration

**FIGURE 2:**
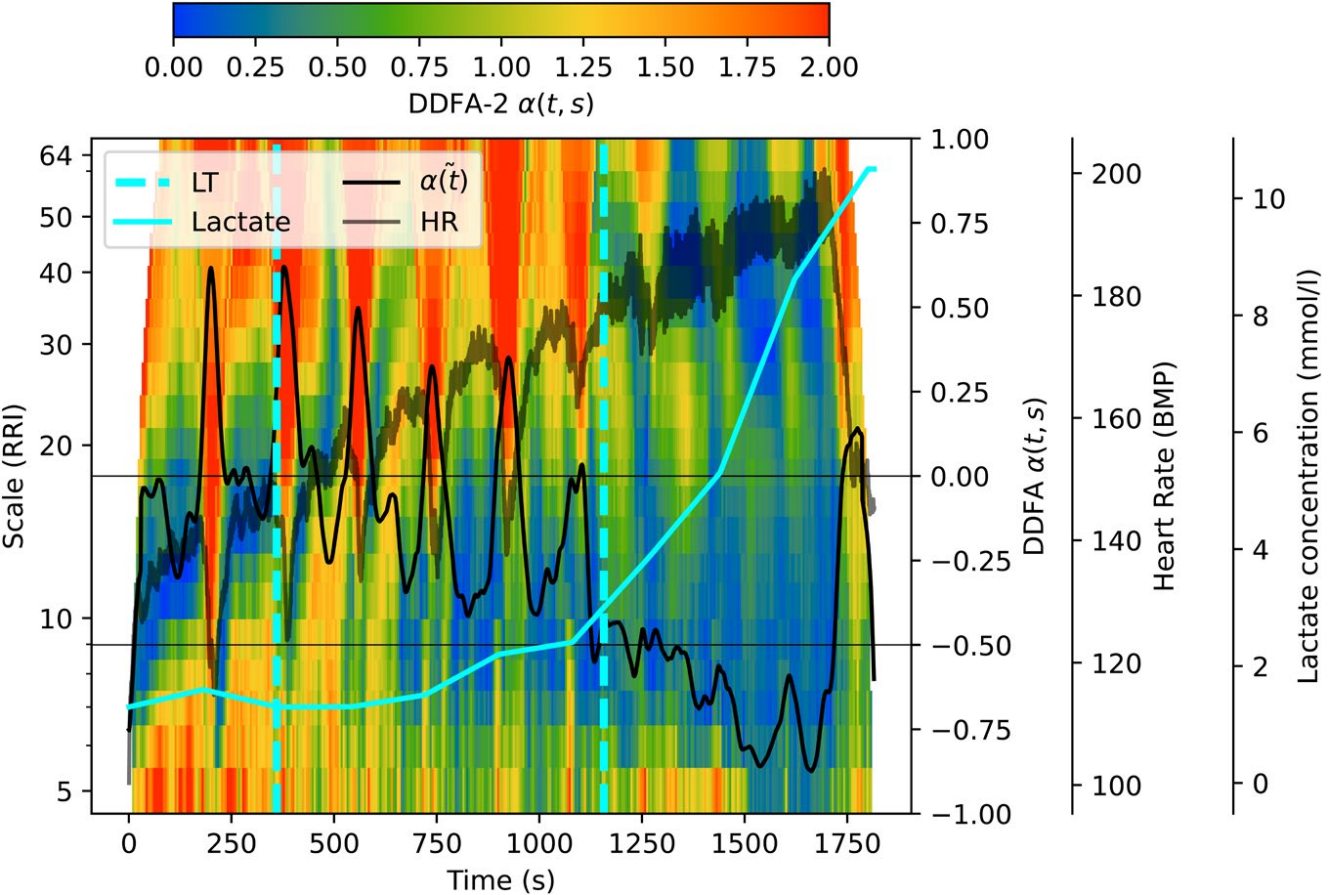
Illustration of the behavior of integrated scaling exponent $\tilde{\alpha }\left(t\right)$ (black line) and the lactate concentration (cyan line) during the exercise as a function of time. The lactate thresholds are plotted with cyan vertical lines, and the gray line corresponds to heart rate during the exercise.

Before comparing the DDFA thresholds with the conventional maximal HR and lactate methods, we study the behavior of the RRIs as a function of the blood lactate concentration. The lactate measurement points are separated by 3 min due to the type of the measurement, so we apply linear interpolation between the data points to study the lactate continuously during the test. An example of an exercise test with lactate concentration is shown in Figure 2. Here, the colormap corresponds to the DDFA scaling exponent *α*(*t*,*s*) as in Figure 1.

As described in Section 2.5.2, the DDFAT_1_ can be estimated in the point, where the integrated and smoothed scaling exponent falls below the zero level. Here, the black line represents the scaling exponent integrated as a function of time $\tilde{\alpha }\left(t\right)$. However, due to the stops where the lactate sample is collected, the $\tilde{\alpha }\left(t\right)$ returns above the zero‐level several times, but is often increased back above the threshold. Unlike $\tilde{\alpha }\left(t\right)$, the lactate concentration does not decrease due to the breaks. Similar behavior is seen around the second DDFA threshold, where the small break causes the $\tilde{\alpha }\left(t\right)$ to increase, thus falling back below the anaerobic level. This implies, that according to DDFA, the test is not fully incremental, but instead the training intensity can be changing around the threshold during the test caused by the recovery during the breaks.

**FIGURE 3:**
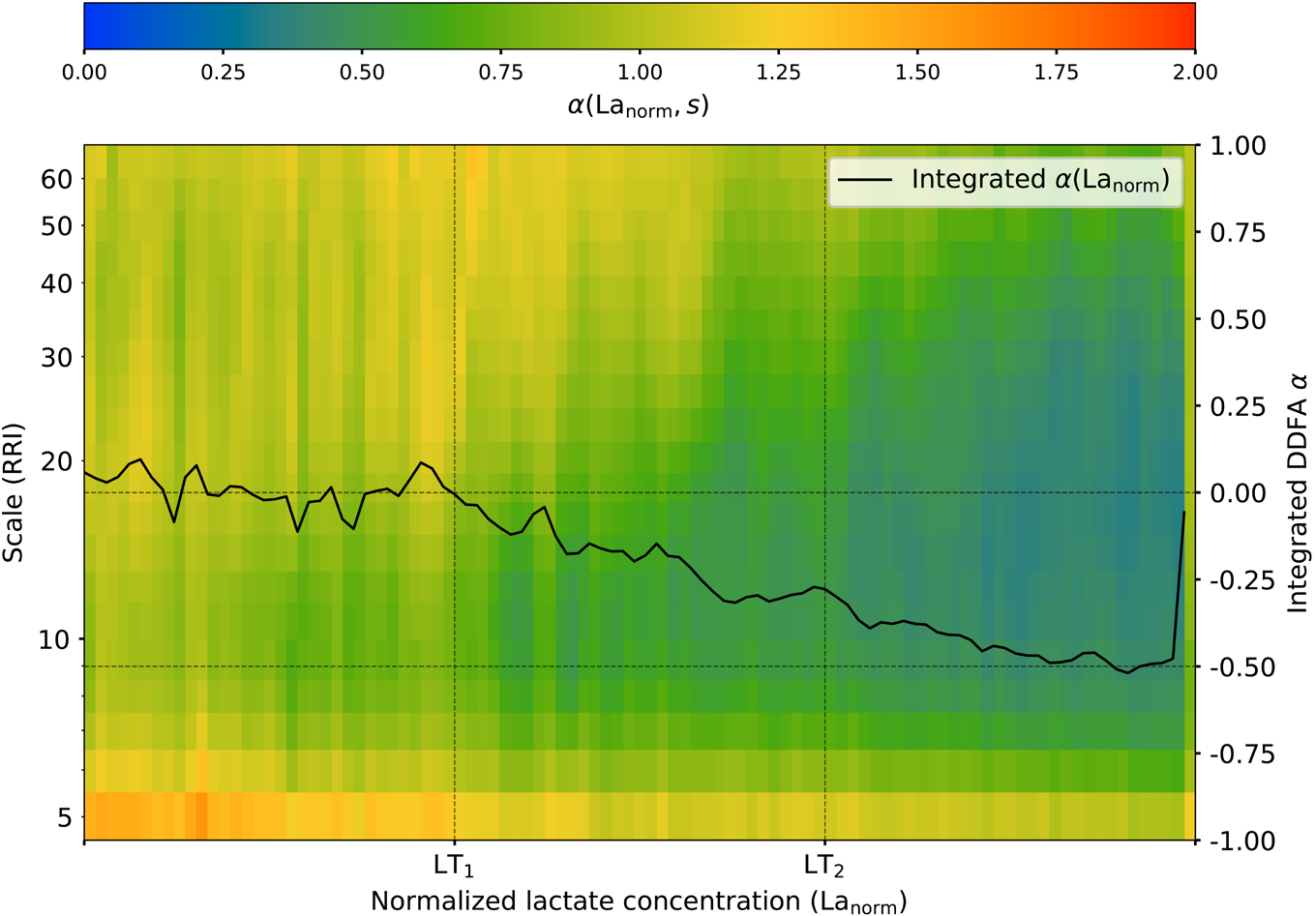
Aggregate plot of the DDFA scaling exponents for all studied subjects as a function of normalized lactate concentration. The black line corresponds to the integrated scaling exponent *α*(La_norm_).

For each subject, the lactate concentration at the lactate thresholds LT_1_ and LT_2_ is different. Therefore, we normalize the lactate concentration for all of the subjects (i) before LT_1_, (ii) between the two lactate thresholds, and (iii) after LT_2_ to uniformly distributed intervals of [0,1]. To study the relationship between lactate concentration and the behavior of the RRIs, we combine the data of all of the studied subjects. In Figure 3 the aggregated DDFA scaling exponent *α*(La_norm_,*s*) is shown as a function of the normalized lactate concentration and scale. The *α*(La_norm_,*s*) is averaged over the scales obtaining the integrated scaling exponent. The *α*(La_norm_,*s*) curve is scaled between [−1,1] to be qualitatively consistent with the threshold determination.

Considering the lactate baseline at *α*(La_norm_ = 0), on average, the integrated *α*(La_norm_) drops below the baseline exactly at LT_1_. The integrated *α*(La_norm_) keeps decreasing when moving towards LT_2_ and crosses the threshold at *α*(La_norm_) = −0.3. In contrast to our threshold method, where the second threshold is determined at the decrease of −0.5 from the baseline, *α*(La_norm_) only reaches −0.5 after the threshold. This is due to the fact, that during the lactate measurement breaks, the lactate concentration is not decreasing in the body, but the HR and the $\tilde{\alpha }\left({\mathrm{HR}}_{\mathrm{bin}}\right)$ distributions are temporarily decreased. Therefore, even after the LT_2_, the training intensity can lower due to the breaks, and the $\tilde{\alpha }\left({\mathrm{HR}}_{\mathrm{bin}}\right)$ can temporarily fall back to the zone between the thresholds, as seen in Figure 2. However, this analysis still exhibits the consistent relationship between the DDFA scaling exponent and the lactate concentration around the thresholds.

### 3.3 Comparison of the methods

First, we calculate Pearson's correlation coefficients *r* with their p‐values and 95% confidence interval lower bounds, and the mean differences (*t*‐test statistic) and their *p*‐values for the different threshold estimation methods against the lactate thresholds with two‐sided *t*‐test. The overall sample size power from the two‐sided *t*‐test is 0.76 for the studied group of 58 subjects. The statistics are shown in Table 2.

**TABLE 2:** Statistical measures for the different methods against the lactate thresholds: Pearson correlation coefficients *r*, their *p*‐values (*p*_corr_), their 95% confidence interval lower bounds (LB), mean differences *μ*_diff_ (BPM) and their *p*‐values (*p*_diff_). The sample size power is 0.76 for the studied set of data.

Threshold	Pearson r	$p_{\text{corr}}$	95% LB	$\mu_{\text{diff}}$	$p_{\text{diff}}$
${\mathrm{HR}}_{\max}^{\text{theor}}$ T_1_	0.31	0.02	0.05	13.19	$10^{-21}$
${\mathrm{HR}}_{\max}^{\text{theor}}$ T_2_	0.47	$10^{-04}$	0.24	11.24	$10^{-19}$
${\mathrm{HR}}_{\max}^{\text{meas}}$ T_1_	0.55	$10^{-06}$	0.34	10.65	$10^{-17}$
${\mathrm{HR}}_{\max}^{\text{meas}}$ T_2_	0.78	$10^{-13}$	0.65	7.24	$10^{-11}$
DDFAT_1_	0.43	$10^{-04}$	0.19	0.92	0.36
DDFAT_2_	0.58	$10^{-06}$	0.38	−3.16	$10^{-03}$

For ${\mathrm{HR}}_{\max}^{\text{theor}}$ Ts compared to LTs, the correlations are moderate (0.3 ≤ *r* ≤ 0.5), and they are statistically significant (*p*_corr_ < 0.05). The means (*μ*_diff_) against both LT_1_ and LT_2_ are the largest for the ${\mathrm{HR}}_{\max}^{\text{theor}}$ method from all of the compared methods (13.19, 11.24 for LT_1_ and LT_2_, respectively). With the thresholds derived from the measured ${\mathrm{HR}}_{\max}^{\text{meas}}$, on the other hand, the correlations are the largest (0.55, 0.78) from the compared methods, but the mean differences *μ*_diff_ (10.65, 7.24) are only slightly better compared to those of the ${\mathrm{HR}}_{\max}^{\text{theor}}$ Ts. Finally, DDFA thresholds yield relatively good correlations (0.43, 0.58) with statistical significance for the both thresholds, and the mean differences are by far the smallest to lactate thresholds between the methods (0.92, −3.16). We want to point out that here larger *p*‐values of the differences *p*_diff_ indicate a smaller mean difference to the lactate thresholds.

**FIGURE 4:**
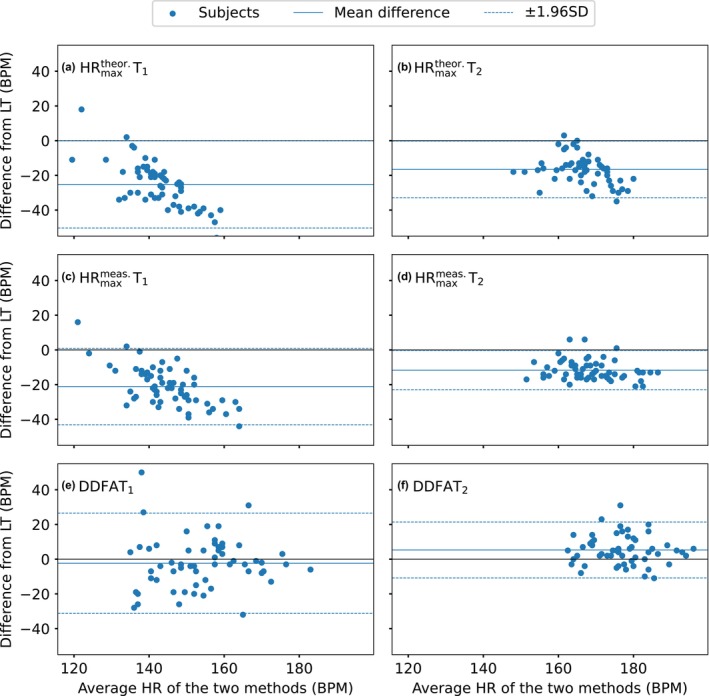
Bland–Altman plots of the differences between (a) ${\mathrm{HR}}_{\max}^{\text{theor}}$ T_1_ and LT_1_, (b) ${\mathrm{HR}}_{\max}^{\text{theor}}$ T_2_ and LT_2_, (c) ${\mathrm{HR}}_{\max}^{\text{meas}}$ T_1_ and LT_1_, (d) ${\mathrm{HR}}_{\max}^{\text{meas}}$ T_2_, and LT_2_, (e) DDFAT_1_ and LT_1_, and (f) DDFAT_2_ and LT_2_. The black solid lines correspond to the difference of 0 BPM, blue solid lines correspond to the mean differences and the blue dashed lines correspond to the 95% limits of agreement (= ± 1.96× standard deviation (SD)) of the studied set of data.

To analyse the head‐to‐head differences between lactate thresholds in more detail for DDFA thresholds and maximal HR thresholds, we utilize Bland–Altman plots. Figure 4 shows the differences of (a) ${\mathrm{HR}}_{\max}^{\text{theor}}$ T_1_, (b) ${\mathrm{HR}}_{\max}^{\text{theor}}$ T_2_, (c) ${\mathrm{HR}}_{\max}^{\text{meas}}$ T_1_, (d) ${\mathrm{HR}}_{\max}^{\text{meas}}$ T_2_, (e) DDFAT_1_, and (f) DDFAT_2_ to the corresponding lactate thresholds LT_1_ and LT_2_. The solid lines correspond to the mean differences to lactate, and the dashed lines correspond to the 95% limits of agreement, which can also be expressed as ±1.96 standard deviation (SD) from the difference.

For both ${\mathrm{HR}}_{\max}^{\text{theor}}$ T_1_ and ${\mathrm{HR}}_{\max}^{\text{theor}}$ T_2_, in Figure 4a,b, there is a systematic bias, and the distributions are considerably below the zero‐level. Here, the mean differences are −25 BPM and −17BPM for the ${\mathrm{HR}}_{\max}^{\text{theor}}$ T_1_and ${\mathrm{HR}}_{\max}^{\text{theor}}$ T_2_, respectively. Similar behavior is noticed in the ${\mathrm{HR}}_{\max}^{\text{meas}}$ thresholds, where the mean differences of the first and second thresholds are −21 BPM and −11 BPM, respectively. Overall, the thresholds derived from ${\mathrm{HR}}_{\max}^{\text{meas}}$ are more consistent with the lactate thresholds compared to the ones derived from ${\mathrm{HR}}_{\max}^{\text{theor}}$. For DDFAT_1_, the mean difference −2 BPM, but there are a few outliers on both sides of the zero level. The limits of agreement are +23 BPM and –27 BPM, which indicates that there is some error on the estimation on the individual level. In Figure 4f, the threshold estimation is consistent with a mean difference of 5 BPM to the lactate thresholds. Here, however, the limits of agreement are considerably smaller than those of DDFAT_1_, also with considerably fewer outliers.

**FIGURE 5:**
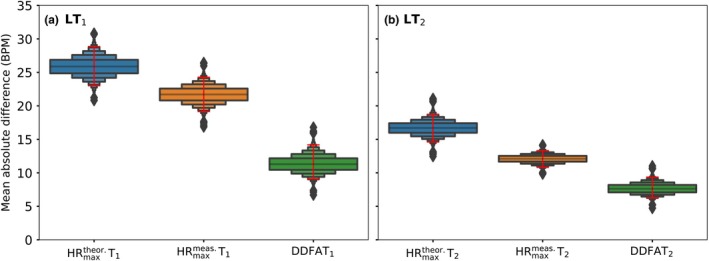
The error of the estimation for each of the compared method for (a) first lactate threshold, and (b) second lactate threshold. The boxenplots correspond to the mean differences of the estimates, and the red lines correspond to the 95% confidence intervals (CI) after bias‐corrected and accelerated (BCa) bootstrapping (10^4^ times).

Next, we consider the mean difference of the head‐to‐head estimations of the thresholds with limits of agreement to measure the total error in the threshold estimation with different methods. In Figure 5 we compare again the thresholds derived from two different maximal HR measures, ${\mathrm{HR}}_{\max}^{\text{theor}}$ and ${\mathrm{HR}}_{\max}^{\text{meas}}$, and the DDFA‐based thresholds to the lactate thresholds. The red lines stand for the 95% BCa confidence intervals, and the boxenplots correspond to the mean differences to lactate thresholds after bootstrapping.

Here, the ${\mathrm{HR}}_{\max}^{\text{theor}}$ Ts have the errors of estimation with respect to both LTs for all of the compared methods. The medians of the boxenplots are the highest (26 BPM and 17 BPM for LT_1_ and LT_2_, respectively), implying that the median error of the mean differences is the largest for the method. The ${\mathrm{HR}}_{\max}^{\text{meas}}$ Ts, on the other hand, have smaller median errors of the mean differences of 21 and 13 BPM for the LT_1_ and LT_2_, respectively. DDFA thresholds have the smallest medians of mean differences for both the LT_1_ (12 BPM) and LT_2_ (10 BPM). This indicates that DDFATs have the best agreement to the lactate thresholds compared to both of the maximal HR‐based methods.

## 4 DISCUSSION

In this study, we validate our previously published method (Kanniainen et al., 2023;  Molkkari & Räsänen, 2023) to determine the aerobic and anaerobic thresholds in running based on the dynamical correlation properties of RRIs detected by DDFA. The method is based on the fact that the DFA short‐scale scaling exponent *α*_1_ is known to decrease during the exercise (Casties et al., 2006; Gronwald et al., 2021; Rogers et al., 2021; Hautala et al., 2003). This fact also applies to the DDFA scaling exponent, which captures the dynamical behavior of the RRI scaling properties as a function of the exercise variables, such as HR (Kanniainen et al., 2023). We analyse the exercise intensity from the DDFA scaling exponents *α*(HR,*s*) for treadmill running tests and calculate the HR values corresponding to LT_1_ and LT_2_. The agreement of our estimates with LT_1_ and LT_2_ is good, and the method shows no systematic bias.

We also evaluated the thresholds derived from maximal heart rate. We calculated the theoretical heart rate values using the conventional formula of HR_max_ = 220 − AGE, but also derived the thresholds from the maximal heart rates that the subjects reached during the test. Between these two methods alone, there was a significant disagreement, demonstrating the importance of a universal method. Apart from this, the age‐dependent formula for maximal HR has been subject to criticism in the field of physiology (Colantonio & Peduti Dal Molin Kiss, 2013; Robergs et al., 2002), but is still being used in several smartwatches for fitness purposes. Furthermore, the maximal heart rate for individual often varies with different types of exercise (Faff et al., 2007). The optimal ratio of HR/HR_max_ for the thresholds is also often subject to individuality based on the fitness level of the athletes. For consistency, we use the same percentages (70% and 85%) as in our earlier study (Kanniainen et al., 2023). In summary, currently there is no universal method to derive the thresholds reliable from the maximal HR for different testing protocols and individuals with different training backgrounds.

In Figure 4, the distributions are more dense; that is, the confidence intervals are closer to the zero‐level for the maximal HR threshold values compared to DDFA threshold values, but there is still a systematic bias on the thresholds compared to lactate. As discussed above, the percentages of the maximal HR value at the thresholds can vary a lot between individuals and studied groups. Therefore, with case‐by‐case optimized percentages, also the HR_max_‐derived thresholds could be improved. However, we want to point out that in (Kanniainen et al., 2023), the same percentage values were used with relatively good results for HR_max_ thresholds. Therefore, there is evidently no agreement across different populations and modes of exercise. On the other hand, qualitatively, there is no systematic bias in either of the DDFA thresholds compared to the lactates.

In this study, we used lactate thresholds as the baseline method for comparison with other measures. Although lactate thresholds are often regarded as the reference standard for analysing AeT and AnT, it is important to note that there is no universally accepted golden standard for determining LT_1_ and LT_2_. The identification of these thresholds is often subjective and ambiguous, as several different methods exist for determining lactate thresholds (Faude et al., 2009; Svedahl & MacIntosh, 2003). Therefore, even when employed by an experienced exercise physiologist, lactate threshold methods should be interpreted with caution. Ventilatory thresholds are frequently used as a benchmark. However, in this study, we chose to rely on lactate thresholds, as they are commonly considered the reference standard in Finland (Keskinen et al., 2018).

Our results demonstrate that, in addition to the reliable outcomes previously shown for cyclo‐ergometer tests in (Kanniainen et al. 2023), the method based on dynamical RR interval correlations also provides consistent and accurate results for running exercise tests conducted with the treadmill testing protocol. Furthermore, the results are consistent throughout the dataset with subjects of considerably different exercise backgrounds. Finally, a significant advantage of the DDFA method is the possibility to use it in real time during exercise to evaluate the exercise intensity continuously with the accumulated data. Therefore, the user could have a real‐time estimate of their current training zone directly from a wearable device, that is, a smartwatch without the need to pause the exercise to do, for example, lactate measurements.

Our study has some limitations. First, the incremental treadmill exercise tests were carefully planned and conducted in a controlled, stable environment. Therefore, it remains to be seen how the method performs in more challenging exercise conditions where the protocol is not strictly adhered to. Additionally, most exercises performed by smartwatch or heart rate monitor users are not incremental to exhaustion but instead occur at varying intensities. Furthermore, our method has only been tested in cycling and running, so further validation is required for other sports disciplines.

Another potential limitation is the data quality obtained from smartwatches and heart rate monitors available on the consumer market. Here, we have used Polar H10 heart rate sensor, which has been shown to yield strong agreement with ECG recordings (Schaffarczyk et al., 2022). It would be valuable to explore whether DDFAT thresholds can be accurately estimated from RR intervals measured by devices such as smartwatches that use photoplethysmography (PPG). Although our threshold estimation method has not yet been applied in such settings, it is important to note that several studies support the reliability of PPG measurements during exercise, showing results comparable to those from ECG recordings (Weiler et al., 2017; Temko, 2017). However, close attention must be paid to signal quality, and implementing a dynamic preprocessing tool for the data stream is essential. This is an area that warrants further validation of the method.

## 5 CONCLUSION

Here we have validated our computational method to estimate the aerobic (AeT) and anaerobic thresholds (AnT) from the dynamical correlation properties of RR intervals with dynamical detrended fluctuation analysis (DDFA). We estimated the DDFA‐based thresholds (DDFAT_1_ and DDFAT_2_) and compared them to the lactate thresholds LT_1_ and LT_2_. Furthermore, we calculated thresholds from theoretical and measured maximal HR, which are often used in the smartwatches and heart rate monitors available in the consumer market. We calculated the thresholds with all of the methods for 58 subjects in an incremental treadmill running test.

We found significant differences in the thresholds derived from the theoretical and measured maximal heart rates alone. The ${\mathrm{HR}}_{\max}^{\text{theor}}$ thresholds were consistently estimated below the lactate thresholds, and there was a systematic bias in the estimation. Also, the ${\mathrm{HR}}_{\max}^{\text{meas}}$ thresholds had a systematic bias in the estimation, and again the thresholds were estimated consistently below the lactate thresholds, even though the agreement to lactate was better than for the theoretical maximal HR estimation. Our DDFAT method yielded reasonable agreement with the lactate thresholds, and there was no systematic bias in the results.

The DDFA‐based method offers a simple and accurate alternative for the estimation of the AeT and AnT, which can be implemented in real‐time monitoring of the training intensity during exercise. The laboratory measurements are tedious and expensive, and our method allows the continuous long‐term monitoring of performance.

## AUTHOR CONTRIBUTIONS

MK: conceptualization, data curation, formal analysis, investigation, methodology, software, validation, visualization, writing—original draft. VLR: conceptualization, data curation, formal analysis, investigation, software, writing—review and editing. TP: conceptualization, formal analysis, investigation, methodology, writing—review and editing. KV: conceptualization, data curation, formal analysis, investigation, writing—review and editing. EH: conceptualization, data curation, investigation, project administration, writing—review and editing. JI: conceptualization, funding acquisition, investigation, project administration, supervision, writing—review and editing. ER: conceptualization, funding acquisition, investigation, project administration, supervision, writing—review and editing. All authors approved the final version of manuscript.

## FUNDING INFORMATION

Finland, Business Finland/Research to Business (R2B) MoniCardi Project (Grant No. 1426/31/2022), The Kalle Kaihari Heart Research Fund of The University of Tampere Foundation, The Finnish Foundation for Cardiovascular Research (Grant No. 230078), Elli and Elvi Oksanen Fund of the Finnish Cultural Foundation Pirkanmaa Regional Fund (Grant No. 50231659), and The European Regional Development Fund (ERDF, R‐00083).

## CONFLICT OF INTEREST STATEMENT

MK, TP, and ER are shareholders of MoniCardi Ltd. focusing on cardiac health assessment. The research was carried out fully independently of the company's involvement, and the company did not influence the study design, data collection, analysis, or interpretation. ER is involved in a pending patent associated to the field of study (Molkkari & Räsänen., 2023 in the manuscript). Other authors declare no conflicts of interest, financial, or otherwise, regarding this study.

## ETHICS STATEMENT

This manuscript was submitted to Physiological Reports. For this study, 71 participants were recruited as a part of a larger study conducted in the University of Jyväskylä. Data collection was performed during an ongoing study project “Performance variation and health monitoring of recreational runners” that monitors training, health, and recovery of recreational runners and active controls through one calendar year. As the corresponding author, I confirm that the study protocol was approved by the ethics committee of the University of Jyväskylä (534/13.00.04.00/2023), and a written informed consent to participate in the study project was obtained from each subject.

## Data Availability

The data is not available publicly due to privacy issues. To discuss the possibility of accessing the data, please contact VLR (vesa.h.laatikainenraussi@jyu.fi).
